# Speech perception in noise in the elderly: interactions between cognitive performance, depressive symptoms, and education^☆^

**DOI:** 10.1016/j.bjorl.2016.03.017

**Published:** 2016-04-27

**Authors:** Laura Maria Araújo de Carvalho, Elisiane Crestani de Miranda Gonsalez, Maria Cecília Martineli Iorio

**Affiliations:** a Universidade Federal de São Paulo (UNIFESP), Programa Pós-graduação em Distúrbios da Comunicação Humana, São Paulo, SP, Brazil; b Faculdade de Ciências Médicas da Santa Casa de São Paulo, São Paulo, SP, Brasil; c Universidade Federal de São Paulo (UNIFESP), Departamento de Fonoaudiologia, São Paulo, SP, Brazil

**Keywords:** Cognition, Depression, Elderly, Speech in noise test, Cognição, Depressão, Idoso, Teste de fala em ruído

## Abstract

**Introduction:**

The difficulty the elderly experience in understanding speech may be related to several factors including cognitive and perceptual performance.

**Objective:**

To evaluate the influence of cognitive performance, depressive symptoms, and education on speech perception in noise of elderly hearing aids users.

**Methods:**

The sample consisted of 25 elderly hearing aids users in bilateral adaptation, both sexes, mean age 69.7 years. Subjects underwent cognitive assessment using the Mini-Mental State Examination and the Alzheimer's Disease Assessment Scale-cognitive and depressive symptoms evaluation using the Geriatric Depression Scale. The assessment of speech perception in noise (S/N ratio) was performed in free field using the Portuguese Sentence List test. Statistical analysis included the Spearman correlation calculation and multiple linear regression model, with 95% confidence level and 0.05 significance level.

**Results:**

In the study of speech perception in noise (S/N ratio), there was statistically significant correlation between education scores (*p* = 0.018), as well as with the Mini-Mental State Examination (*p* = 0.002), Alzheimer's Disease Assessment Scale-cognitive (*p* = 0.003), and Geriatric Depression Scale (*p* = 0.022) scores. We found that for a one-unit increase in Alzheimer's Disease Assessment Scale-cognitive score, the S/N ratio increased on average 0.15 dB, and for an increase of one year in education, the S/N ratio decreased on average 0.40 dB.

**Conclusion:**

Level of education, cognitive performance, and depressive symptoms influence the speech perception in noise of elderly hearing aids users. The better the cognitive level and the higher the education, the better is the elderly communicative performance in noise.

## Resumo

**Introdução:**

A dificuldade na compreensão de fala dos idosos pode estar relacionada a vários fatores, como o desempenho cognitivo e perceptual.

**Objetivo:**

Avaliar a influencia do desempenho cognitivo, sintomas depressivos e escolaridade no reconhecimento de fala no ruído de idosos usuários de próteses auditivas.

**Método:**

A amostra constituiu-se de 25 idosos usuários de próteses auditivas em adaptação bilateral, de ambos os sexos e idade média de 69,7 anos. Os indivíduos foram submetidos à avaliação cognitiva por meio do Mini-Exame do Estado Mental (MEEM) e a Escala de Avaliação da Doença de Alzheimer-Cognitiva (ADAS-Cog) e avaliação de sintomatologia depressiva por meio da Escala de Depressão Geriátrica (EDG). Já a pesquisa do reconhecimento de fala no ruído (relação S/R) foi realizada, em campo livre, por meio do teste Lista de Sentenças no Português (LSP). A análise estatística incluiu o cálculo de correlação de Spearman e modelo de regressão linear múltiplo, sendo adotado coeficiente de confiança de 95% e nível de significância de 0,05.

**Resultados:**

No estudo do reconhecimento de sentenças no ruído (relação S/R) houve correlação, com significância estatística, entre a escolaridade (*p* = 0,018); assim como, com os escores dos testes MEEM (*p* = 0,002); o Adas-Cog (*p* = 0,003) e o EDG (*p* = 0,022). Observou-se que, para um aumento de uma unidade no escore do Adas-Cog, a relação S/R aumenta, em média; 0,15 dB e para um aumento de um ano na escolaridade, a relação S/R diminui, em média; 0,40 dB.

**Conclusão:**

O nível de escolaridade, o desempenho cognitivo e sintomas depressivos influenciam o reconhecimento de fala no ruído de idosos usuários de prótese auditiva. Quanto melhor o nível cognitivo e maior a escolaridade melhor é o desempenho comunicativo do idoso no ruído.

## Introduction

Auditory function deterioration and cognitive decline are conditions commonly found in the elderly population and may compromise the aging process. Hearing loss due to aging is characterized by decreased hearing sensitivity mainly for high frequency sounds, and speech perception difficulties.^1^, ^2^, ^3^

The use of hearing aids is a primary means in the rehabilitation of hearing loss.^4^ However, due to the combination of peripheral and central changes, elderly people often do not achieve satisfactory improvement in speech recognition in competitive noise situations using amplification.

Several studies have shown that the understanding of sentences in noise is impaired in patients with dementia.^5^, ^6^ This deficit may be attributed to the difficulty in processing grammatical and/or semantic information, as well as to a higher cognitive demand required for decoding and understanding the speech signal in noise.^7^

Cognitive abilities, such as attention, memory and language, are involved in the process of speech detection, discrimination, understanding, and organization. Understanding the interaction of cognitive functions with sensory functions can assist in the rehabilitation of the elderly with difficulty in adapting to hearing aids, as well as guiding the technological advancement in the field of prosthetics.^8^, ^9^

It is known that a cognitive impairment may be associated with history of depressive symptoms. The most frequent cognitive impairments in depressed elderly are the executive function, intentional deficits, and reduced processing speed. Because depression can simulate dementia,^10^, ^11^ the investigation of depressive symptoms in elderly patients with hearing aids is appropriate since the presence of these symptoms can interfere negatively in the communication process.

Based on these considerations, our hypothesis was that the speech perception in noise in elderly hearing aids users is impaired when associated with cognitive decline and presence of depressive symptoms. Thus, this study aimed to evaluate the effect of cognitive and depressive factors on the speech perception in noise performance of elderly hearing aids users.

## Methods

### Ethical considerations

This is an experimental study with non-probabilistic convenience sample. The study was approved by the Research Ethics Committee of the institution, and participants were informed about the objectives and methodology of the proposed study, agreed to participate voluntarily and gave written informed consent.

### Selection of subjects and case series

The sample was selected according to the following eligibility criteria:

• Age equal to or greater than 60 years;
• Have sensorineural, symmetrical, mild to moderately severe acquired hearing loss, according to the Lloyd and Kaplan classification, 1978;
• User hearing aids in bilateral adaptation, identical models, in-the-ear or behind-the-ear hearing aid;
• Effective user of amplification for at least three months (after acclimatization);
• Absence of obvious impairment (neurological, speech and/or verbal fluency).

According to the eligibility criteria, 25 individuals were called by phone to participate in this study, 9 males and 16 females, aged between 60 and 85 years (mean 69.7 years).

### Procedures

All patients underwent assessment of cognitive and depressive aspects, as well as the signal-to-noise ratio (S/N) of the Portuguese Sentence List test. These procedures were performed in a single evaluation session lasting about 90 min.

### Cognitive assessment

The cognitive assessment was performed using the Mini-Mental State Examination (MMSE) and Alzheimer's Disease Assessment Scale (ADAS-Cog) tests.

MMSE is a screening test used to measure cognitive function; in Brazil, it was adapted by Bertolucci et al.^12^ The test evaluates eight cognitive parameters divided into seven categories: temporal and spatial orientation, short-term memory and recall of words, calculation, praxis, language and visuospatial abilities. The test score ranges from 0 to 30 points. The lower the score, the greater the chance of the individual to present changes in cognitive ability. In this study, we used the scoring classification of Brucki et al.^13^

In this study, the cognitive session of Alzheimer's disease Assessment Scale (ADAS-Cog) was also used, which is composed of 11 items that assess memory (recognition and recall), language (speech and understanding), and praxis (copy and ideomotor). The score was classified according to the education of the subject, and those outside the range equivalent to the mean plus two standard deviations were considered abnormal.^14^

It should be noted that these scales were used to rate the cognitive performance of individuals in normal or abnormal category, according to their education. No participant had a diagnosis of Alzheimer's disease.

### Depression screening

For depressive symptoms evaluation, the shorter version of the Geriatric Depression Scale (GDS-15) was applied, consisting of 15 questions with alternative closed answers (“yes” or “no”). For analysis of results, the scoring criteria suggested by Almeida were employed,^11^ with scores above 5 considered as abnormal.

### Signal-to-noise ratio (S/N) of the Portuguese Sentence List test

To evaluate the S/N ratio, the Portuguese Sentences List test^15^ was used, which consists of lists with 10 sentences each, and a background noise of speech spectrum. In this assessment, the test application was in free field in an acoustically treated environment, with the subject positioned at 1 m from the sound source on condition 0° azimuth, that is, in front of the subject. Sentence presentation followed the ascending-descending strategy.^16^ Noise level was kept constant during sentence presentation, only modifying the presentation intensity of sentences. The initial S/N ratio was established at +5 dB. To calculate the S/N ratio, the average value of SRTN was subtracted from the noise intensity level. Thus, the S/N ratio corresponded to the difference in dB between the value of SRTN and competitive noise.

### Statistical analysis

To study the correlation between the tests and S/N ratio with Age, Education, and PSAD usage time; scatter diagrams were constructed and Spearman correlation coefficients were calculated.^17^ The same procedures were used in the study of correlation between tests (two by two) and between tests and S/N ratio. A regression model^17^ was adjusted, having as variable the response to S/N ratio and as possible explanatory variables the tests and education. Forward stepwise procedure was used in the selection of the explanatory variables. Mean scores in the four tests were also estimated by range, adopting a 95% confidence level. In hypothesis testing, 0.05 significance level was set and the significant values were graphed with an asterisk (*).

## Results

Initially, an analysis of descriptive statistics was performed for Age, Education, and PSAD usage time (Table 1).

**Table 1:** Descriptive statistics for age (years), education (years), and PSAD usage time (months).

Variable	*n*	Mean	Standard deviation	Minimum	Median	Maximum
Age	25	69.7	7.6	60	67	85
Education	25	4.5	3.1	0	4	11
PSAD time	25	10.2	6.5	3	7	27

PSAD, personal sound amplification devices.

A comparative study was conducted between sentence recognition in noise (S/N ratio) and the variables hearing aids usage time, education, and age. There was a negative statistically significant correlation between S/N ratio and education; that is, the higher the education, the lower the S/N ratio (Table 2).

**Table 2:** Values of Spearman correlation coefficients16 between S/N ratio and PSAD time, education, and age.

S/N ratio	R	P
PSAD time	−0.13	0.536
Education	−0.47	0.018*
Age	−0.22	0.286

PSAD, personal sound amplification devices.

Table 3 shows the correlation between the PSL S/N ratio and the scores of MMSE, ADAS-Cog, and GDS. There was a significant correlation between the S/N ratio and studied tests. Thus, the higher the MMSE score, the lower the ADAS-Cog and GDS scores, the lower the S/N ratio for the PSL test.

**Table 3:** Values of Spearman correlation coefficients16 between S/N ratio and MMSE, ADAS-Cog, and GDS.

	MEEM	ADAS-cog	GDS
*S/N ratio*			
R	−0.57	0.58	0.46
P	0.003*	0.002*	0.022*

A multiple linear regression model was conducted from the relationship between the S/N ratio and ADAS-Cog and education variables (Table 4).

**Table 4:** Multiple linear regression model between S/N ratio and ADAS-Cog and education variables.

Expected S/N ratio = −0.037 + 0.15 × ADAS-Cog − 0.40 × Education

From the ADAS-cog and education coefficients in the adjusted model, we found that for a one-unit increase in ADAS-Cog score, the S/N ratio increases on average 0.15 dB and for an increase of one year in education, the S/N ratio decreases on average 0.40 dB.

## Discussion

Understanding speech is most often impaired by competitive noise. Complaints of difficulties in understanding speech in noisy environments among elderly hearing aids users are becoming more common.^18^, ^19^ In this study, we chose to use phrases recognition tests with the presence of a competitive stimulus (noise) in free field, in order to evaluate these individuals, simulating communication conditions closer to those found in everyday life.

The elderly subjects were randomly selected from a concession service of hearing aids in the metropolitan city. The population looking for that service consists mostly of low income and low education patients, reflecting the socioeconomic profile of the majority of elderly people living in developing countries. In the current sample, there was a low level of education among the participants and, moreover, the S/N ratio obtained in the PSL test tend to increase the lower the level of education, demonstrating that older people with less education have worse performance in difficult listening conditions.

It is important to note that a low educational level can directly affect the performance in cognitive tests, as reported by Bertolucci et al.^12^ Even in people who had no evidence of cognitive impairment, the lower the educational level, the lower the scores on cognitive tests.^12^, ^13^, ^20^, ^21^

According to Pichora-Fuller,^22^ in order to better understand the influence of hearing on the participation of daily life activities of an individual, one should consider the variable age with the differences in cognitive and perceptual performance. Difficulties in speech understanding are increased by factors such as noise and memory. Moreover, the significance of these processes will depend on socio-emotional characteristics presented by each individual.

The present study found correlation between the scores of the PSL S/N ratio and MMSE and ADAS-Cog cognitive tests, and the lower the cognitive level, the worse the hearing performance of the elderly in noise. Speech recognition in noise is a task that requires the use of memory, attention, and the closing ability, because the listener needs to identify the message distorted by noise and seek information of speech in memory.^23^, ^24^ The study by Pichora Fuller et al.^25^ showed that the working memory capacity is reduced when the noise level is increased relative to the signal.

Lunner,^26^ Akeroyd,^27^ Gates et al.,^24^ Miranda,^28^ and Besser et al.^7^ found correlations between cognitive performance and the ability to recognize speech in noise, and the individuals with better performance on cognitive tests showed better results in speech recognition task. Several studies in the literature indicated that speech recognition in noise requires a demand of cognitive skills, which are in decline in the elderly.^26^, ^27^, ^29^, ^30^, ^31^, ^32^

From the relationship between the S/N ratio and ADAS-Cog and education variables, a multiple linear regression model was developed. The other variables did not present significant additional contribution to the selected ones to explain the S/N ratio. Therefore, it is possible to predict the S/N ratio in which the elderly identifies 50% of the sentences from the ADAS-Cog score and educational level.

The regression model proposed in this study is a tool that can and should be used in the clinic, especially when there are no technological resources that enable the application of speech tests in the presence of competitive noise. From the ADAS-cog and education coefficients in the adjusted model, it is concluded that for a one-unit increase in ADAS-cog score, S/N ratio increases on average 0.15 dB, and for an increase of one year in education, S/N ratio decreases on average 0.40 dB.

In addition to cognitive deficits, depression is also one of the most common health problems in the elderly.^33^, ^34^, ^35^ Depression is a common symptom in the elderly with hearing loss due to functional limitations that this deprivation causes in daily life.^36^, ^37^, ^38^ It is known that depression interferes significantly in the cognitive performance of the patient.^39^

In the current sample, a correlation was found between depressive symptoms and speech in noise recognition. Depressed elderly may have changes in executive function, attention deficits, and decreased processing speed^10^; it is believed that these changes may consequently impair the communicative performance of elderly hearing aids users in difficult listening environments.

From the findings of this study, it is believed that the application of a speech in noise recognition test can be an important tool to help monitor the benefits of the adaptation to hearing aids in the elderly. In the examination of the elderly population with hearing loss, it is difficult to find completely healthy individuals, so it is appropriate to investigate the influence of factors commonly found in this age group that may negatively impact the auditory rehabilitation process, such as cognitive decline and depressive symptoms.

## Conclusion

Level of education, cognitive performance, and depressive symptoms influence the recognition of speech in noise by elderly hearing aid users. The higher the cognitive and educational levels, the better was the speech recognition in noise performance in the elderly hearing aid users.

## Conflicts of interest

The authors declare no conflicts of interest.

## References

[1] Bogardus S.T., Yueh B., Shekelle P.G. (2003). Screening and management of adult hearing loss in primary care: clinical applications. JAMA.

[2] Marques A.C.O., Kozlowski L., Marques J.M. (2004). Reabilitação auditiva no idoso. Rev Bras Otorrinolaringol.

[3] Kooper H., Teixeira A.R., Dorneles S. (2009). Desempenho Cognitivo em um Grupo de Idosos: Influência de Audição, Idade, Sexo e Escolaridade. Arq Int Otorrinolaringolyngol.

[4] Barros P.F.S., Queiroga B.A.M. (2006). As dificuldades encontradas no processo de adaptação de aparelho de amplificação sonora individual em indivíduos idosos. Rev CEFAC.

[5] Grossman M., Rhee J. (2001). Cognitive resources during sentence processing in Alzheimer's disease. Neuropsychologia.

[6] Ranjan R., Bhat J., Kumar U.A. (2011). Cognition and speech perception in noise. Lang India.

[7] Besser J., Zekveld A.A., Kramer S.E., Ronnberg J., Festen J.M. (2012). New measures of masked text recognition in relation to speech in noise perception and their associations with age and cognitive abilities. J Speech Hear Res.

[8] Pichora-Fuller M.K., Singh G. (2006). Effects of age on auditory and cognitive processing: implications for hearing aid fitting and audiologic rehabilitation. Trends Amplif.

[9] Humes L.E. (2007). The contributions of audibility and cognitive factors to the benefit provided by amplified speech to older adults. J Am Acad Audiol.

[10] Ferrari J.F., Dalacorte R.R. (2007). Uso da escala de depressão geriátrica de yesavage para avaliar a prevalência de depressão em idosos hospitalizados. Sci Med.

[11] Almeida O.P., Almeida S.A. (1999). Confiabilidade da versão brasileira da escala de depressão em geriatria (GDS) versão reduzida. Arq Neuro-Psiquiatr.

[12] Bertolucci P.H.F., Brucki S.M.D., Campacci S.R., Juliano Y. (1994). O Mini-exame do estado mental em uma população geral. Arq Neuropsiquiatr.

[13] Brucki S.M.D., Nitrini R., Caramelli P., Bertolucci P.H., Okamoto I.H. (2003). Sugestões para o uso do mini-exame do estado mental no Brasil. Arq Neuro-Psiquiatr.

[14] Schultz R.R., Siviero M.O., Bertolucci P.H. (2001). The cognitive subscale of the alzheimer disease assessment scale in a Brazilian sample. Braz J Med Biol Res.

[15] Costa M.J. (1998).

[16] Levitt H., Rabiner L.R. (1967). Binaural release from masking for speech and gain in intelligibility. J Acoust Soc Am.

[17] Fisher L.D., van Belle G. (1993).

[18] Helfera K.S., Freyman R.L. (2014). Stimulus and listener factors affecting age-related changes in competing speech perception. J Acoust Soc Am.

[19] Gosselin P.A., Gagne J.P. (2011). Older adults expend more listening effort than young adults recognizing speech in noise. J Speech Lang Hear Res.

[20] Almeida O.P. (1998). Mini exame do estado mental e o diagnóstico de demência no Brasil. Arq Neuro-Psiquiatr.

[21] Lourenço R.A., Veras R. (2006). Mini-Exame do Estado Mental: características psicométricas em idosos ambulatoriais. Rev Saúde Pública.

[22] Pichora-Fuller M.K., Souza P.E. (2003). Effects of aging on auditory processing of speech. Int J Audiol.

[23] Anderson S., White-Schwoch T., Parbery-Clark A., Kraus N. (2013). A dynamic auditory-cognitive system supports speech-in-noise perception in older adults. Hear Res.

[24] Gates G.A., Feeney M.P., Mills D. (2008). Cross-sectional age-changes of hearing in the elderly. Ear Hear.

[25] Pichora-Fuller M.K., Schneider B.A., Daneman M. (1995). How young and old adults listen to and remember speech in noise. J Acoust Soc Am.

[26] Lunner T. (2003). Cognitive function in relation to hearing aid use. Int J Audiol.

[27] Akeroyd M.A. (2008). Are individual difference in speech reception related to individual difference in cognitive ability? A survey of twenty experimental studies with normal and hearing-impaired adults. Int J Audiol.

[28] Miranda E.C. (2012).

[29] Schneider B., Speranza F., Pichora-Fuller M.K. (1998). Age-related changes in temporal resolution: envelope and intensity effects. Can J Exp Psychol.

[30] Pichora-Fuller M.K. (2003). Cognitive aging and auditory information processing. Int J Audiol.

[31] Gordon-Salant S., Yeni-Komshian G., Fitzgibbons P.J. (2008). The role of temporal cues in word identification by younger and older adults: effects of sentence context. J Acoust Soc Am.

[32] Stenfelt S., Rönnerberg J. (2009). The signal-cognition interface: interactions between degraded auditory signals and cognitive processes. Scand J Psychol.

[33] Vinkers D.J., Gussekloo J., Stek M.L., Westendorp R.G.J., Vander Mast R.C. (2004). Temporal relation between depression and cognitive impairment in old age: prospective population based study. BMJ.

[34] Nobrega I.R.A.P., Leal M.C.C., Marques A.P.O., Vieira J.C.M. (2015). Fatores associados à depressão em idosos institucionalizados: revisão integrativa. Saúde debate.

[35] Paulo D.L.V., Yassuda M.S. (2010). Queixas de memória de idosos e sua relação com escolaridade, desempenho cognitivo e sintomas de depressão e ansiedade. Rev Psiquiatr Clín.

[36] Tesch-Römer C. (1997). Psychological effects of hearing aid use in older adults. J Gerontol.

[37] Meister H., Lausberg I., Kiessling J., von Wedel H., Walger M. (2002). Identifying the needs of elderly, hearing-impaired persons: the importance and utility of hearing aid attributes. Eur Arch Otorhinolaryngol.

[38] Hidalgo J.L.T., Gras C.B., Lapeira J.T., Verdejo M.A.L., Campo J.M.C., Rabada F.E. (2009). Functional status of elderly people with hearing loss. Arch Gerontol Geriatr.

[39] Almeida P., Jansen K., Köhler C.A., Pinheiro R.T., Silva R.A., Bonini J.S. (2012). Working and short-term memories are impaired in postpartum depression. J Affect Disord.

